# Supplementary material for: Heparin-based hydrogel scaffolding alters the transcriptomic profile and increases the chemoresistance of MDA-MB-231 triple-negative breast cancer cells
Source: Biomater Sci. 2020 Feb 13;8(10):2786–96. doi: 10.1039/c9bm01481k (PMC7497406; doi:10.1039/c9bm01481k)
Supplement: Supplementary file 2 [file BM-008-C9BM01481K-s002.zip › Supplementary File 4/HP-BhydrogelvControl/Pathways/my_analysis.Gsea.1545201079907/gsea_report_for_HYD_1545201079907.html]

Report for HYD 1545201079907 [GSEA]

| GS  follow link to MSigDB | GS DETAILS | SIZE | ES | NES | NOM p-val | FDR q-val | FWER p-val | RANK AT MAX | LEADING EDGE || 1 | HALLMARK\_MYOGENESIS | Details ... | 135 | 0.41 | 1.64 | 0.001 | 0.028 | 0.069 | 4784 | tags=44%, list=25%, signal=58% |
| 2 | HALLMARK\_WNT\_BETA\_CATENIN\_SIGNALING | Details ... | 35 | 0.47 | 1.51 | 0.023 | 0.068 | 0.295 | 3877 | tags=40%, list=20%, signal=50% |
| 3 | HALLMARK\_KRAS\_SIGNALING\_DN | Details ... | 87 | 0.38 | 1.41 | 0.039 | 0.128 | 0.623 | 6006 | tags=47%, list=31%, signal=68% |
| 4 | HALLMARK\_APICAL\_JUNCTION | Details ... | 147 | 0.33 | 1.32 | 0.040 | 0.221 | 0.905 | 3805 | tags=26%, list=20%, signal=32% |
| 5 | HALLMARK\_ANGIOGENESIS | Details ... | 23 | 0.41 | 1.21 | 0.223 | 0.423 | 1.000 | 2317 | tags=26%, list=12%, signal=30% |
| 6 | HALLMARK\_APICAL\_SURFACE | Details ... | 29 | 0.36 | 1.08 | 0.347 | 0.783 | 1.000 | 1524 | tags=17%, list=8%, signal=19% |
| 7 | HALLMARK\_MYC\_TARGETS\_V2 | Details ... | 56 | 0.31 | 1.05 | 0.378 | 0.795 | 1.000 | 4859 | tags=32%, list=25%, signal=43% |
| 8 | HALLMARK\_NOTCH\_SIGNALING | Details ... | 28 | 0.32 | 0.98 | 0.474 | 0.942 | 1.000 | 4966 | tags=32%, list=26%, signal=43% |
| 9 | HALLMARK\_MITOTIC\_SPINDLE | Details ... | 189 | 0.24 | 0.97 | 0.525 | 0.875 | 1.000 | 5169 | tags=32%, list=27%, signal=43% |
| 10 | HALLMARK\_TGF\_BETA\_SIGNALING | Details ... | 50 | 0.28 | 0.93 | 0.581 | 0.919 | 1.000 | 3024 | tags=22%, list=16%, signal=26% |
| 11 | HALLMARK\_TNFA\_SIGNALING\_VIA\_NFKB | Details ... | 178 | 0.22 | 0.91 | 0.681 | 0.894 | 1.000 | 6358 | tags=36%, list=33%, signal=53% |
| 12 | HALLMARK\_UV\_RESPONSE\_DN | Details ... | 129 | 0.23 | 0.89 | 0.683 | 0.859 | 1.000 | 4227 | tags=23%, list=22%, signal=30% |
| 13 | HALLMARK\_P53\_PATHWAY | Details ... | 179 | 0.20 | 0.81 | 0.888 | 0.974 | 1.000 | 5374 | tags=31%, list=28%, signal=43% |
| 14 | HALLMARK\_ESTROGEN\_RESPONSE\_EARLY | Details ... | 170 | 0.18 | 0.73 | 0.966 | 1.000 | 1.000 | 4443 | tags=22%, list=23%, signal=28% |
| 15 | HALLMARK\_HEDGEHOG\_SIGNALING | Details ... | 26 | 0.20 | 0.59 | 0.968 | 0.996 | 1.000 | 6979 | tags=46%, list=36%, signal=72% |
Table: Gene sets enriched in phenotype **HYD (2 samples) **[plain text format]****
